# Costs of Influenza Illness and Acute Respiratory Infections by Household Income Level: Catastrophic Health Expenditures and Implications for Health Equity

**DOI:** 10.1111/irv.70059

**Published:** 2025-01-09

**Authors:** Natalie Wodniak, Radhika Gharpure, Luzhao Feng, Xiaozhan Lai, Hai Fang, Jianmei Tian, Tao Zhang, Genming Zhao, Fernando Salcedo‐Mejía, Nelson J. Alvis‐Zakzuk, Jorge Jara, Fatimah Dawood, Gideon O. Emukule, Linus K. Ndegwa, I‐Ching Sam, Tsogt Mend, Baigalmaa Jantsansengee, Stefano Tempia, Cheryl Cohen, Sibongile Walaza, Wanitchaya Kittikraisak, Arthorn Riewpaiboon, Kathryn E. Lafond, Nelly Mejia, William W. Davis

**Affiliations:** ^1^ Influenza Division Centers for Disease Control and Prevention Atlanta Georgia USA; ^2^ Thailand Ministry of Public Health‐U.S. Centers for Disease Control and Prevention Collaboration Nonthaburi Thailand; ^3^ School of Population Medicine & Public Health, Chinese Academy of Medical Sciences Peking Union Medical College Beijing China; ^4^ Department of Health Policy and Management, School of Public Health Peking University Beijing China; ^5^ China Center for Health Development Studies Peking University Beijing China; ^6^ Health Science Center – Chinese Center for Disease Control and Prevention Joint Research Center for Vaccine Economics Peking University Beijing China; ^7^ Children's Hospital of Soochow University Suzhou China; ^8^ School of Public Health Fudan University Shanghai China; ^9^ ALZAK Foundation Cartagena Colombia; ^10^ Department of Health Sciences Universidad de la Costa Barranquilla Colombia; ^11^ Post‐Graduation Program in Epidemiology, School of Public Health Univsersity of São Paulo São Paulo Brazil; ^12^ Pan American Health Organization Washington DC USA; ^13^ Influenza Division Centers for Disease Control and Prevention Nairobi Kenya; ^14^ Department of Medical Microbiology, Faculty of Medicine Universiti Malaya Kuala Lumpur Malaysia; ^15^ National Influenza Center National Center for Communicable Diseases Ulaanbaatar Mongolia; ^16^ Center for Respiratory Diseases and Meningitis National Institute for Communicable Diseases Johannesburg South Africa; ^17^ School of Public Health, Faculty of Health Sciences University of the Witwatersrand Johannesburg South Africa; ^18^ Influenza Division Centers for Disease Control and Prevention Pretoria South Africa; ^19^ Mahidol University Nakhon Pathom Thailand; ^20^ Global Immunization Division Centers for Disease Control and Prevention Atlanta Georgia USA

**Keywords:** influenza, cost of illness, health equity, catastrophic health expenditure, acute respiratory infection, household income

## Abstract

**Background:**

Seasonal influenza illness and acute respiratory infections can impose a substantial economic burden in low‐ and middle‐income countries (LMICs). We assessed the cost of influenza illness and acute respiratory infections across household income strata.

**Methods:**

We conducted a secondary analysis of data from a prior systematic review of costs of influenza and other respiratory illnesses in LMICs and contacted authors to obtain data on cost of illness (COI) for laboratory‐confirmed influenza‐like illness and acute respiratory infection. We calculated the COI by household income strata and calculated the out‐of‐pocket (OOP) cost as a proportion of household income.

**Results:**

We included 11 studies representing 11 LMICs. OOP expenses, as a proportion of annual household income, were highest among the lowest income quintile in 10 of 11 studies: in 4/4 studies among the general population, in 6/7 studies among children, 2/2 studies among older adults, and in the sole study for adults with chronic medical conditions. COI was generally higher for hospitalizations compared with outpatient illnesses; median OOP costs for hospitalizations exceeded 10% of annual household income among the general population and children in Kenya, as well as for older adults and adults with chronic medical conditions in China.

**Conclusions:**

The findings indicate that influenza and acute respiratory infections pose a considerable economic burden, particularly from hospitalizations, on the lowest income households in LMICs. Future evaluations could investigate specific drivers of COI in low‐income household and identify interventions that may address these, including exploring household coping mechanisms. Cost‐effectiveness analyses could incorporate health inequity analyses, in pursuit of health equity.

## 1 Background

Seasonal influenza inflicts substantial economic burden on individuals, healthcare systems, and societies in low‐ and middle‐income countries (LMICs) [1, 2, 3]. This burden includes direct medical and nonmedical costs to households and health systems, primarily driven by hospitalizations, as well as indirect costs from productivity losses, and broader impacts to the economy through effects on trade and economic development [2]. A recent systematic review estimated that the total annual cost of influenza illness in LMICs could represent up to 0.3%–7.2% of the national health expenditure [1].

Influenza illness episodes may especially impact lower income households, which could be more vulnerable to the impact of unexpected out‐of‐pocket (OOP) costs for healthcare. Catastrophic health expenditures (CHEs), or health spending that requires a household to reduce expenditures on items needed for basic subsistence to pay for healthcare, can result in financial impoverishment [4, 5] and are commonly defined as costs exceeding 10%, 25%, or 40% of basic annual household consumption [5, 6, 7]. Although the 10% threshold is commonly used [6], studies of CHEs frequently present results using multiple thresholds, due to the setting‐ and household‐specific nuances of assessing financial hardship from paying for health [8]. CHEs are a substantial problem: an estimated 12% of households globally, comprising 808 million people, incurred catastrophic health costs (using the 10% definition) in 2010 [6]. The United Nations' Sustainable Development Goals 3.8.2 and 1.1.1 recognize the impact of health expenditures on household poverty and aim to reduce the number of households with CHEs and the number of households driven into poverty by healthcare costs [7].

The global contribution of influenza illness to CHEs is unknown. To estimate the impact of influenza‐associated illnesses on household finances, assess inequalities in the economic burden of influenza, and assess costs by household income strata, we conducted a secondary analysis of data from studies on the cost of influenza in LMICs. We calculated, by household income strata, total costs and OOP costs for influenza illness and acute respiratory infections. We also calculated OOP costs as a proportion of household income as a proxy for assessing the potential for influenza and acute respiratory infections to cause CHEs.

## 2 Methods

### 2.1 Data Sources

We sought published studies on influenza cost of illness (COI) that included household income data. We reviewed studies identified from a recent systematic review by two coauthors of this study (RG and KEL) on economic evaluations for influenza in LMICs, registered at PROSPERO (international prospective register of systematic reviews) under protocol number CRD42022304803 [1]. In brief, the review described a systematic search using Medline, Embase, Cochrane Library, CINAHL, and Scopus in January 2022 and in October 2023 and included studies with a publication year of 2012 through 2023. Search terms were a combination of the following key words: “influenza” AND “cost” OR “economic”; full methods of the systematic review are previously published [1].

Studies in any language from the systematic review were considered eligible for inclusion in this study if they (1) reported COI for laboratory‐confirmed influenza virus infection, influenza‐like illness (ILI), severe acute respiratory infection (SARI), or acute respiratory infection (ARI) and (2) reported household income data. The original systematic review excluded COI studies that used an ARI case definition; however, those studies were retrieved from screening and considered eligible for inclusion in our analysis. Additionally, unpublished COI data from analyses supported by the US Centers for Disease Control and Prevention (CDC) Influenza Division were eligible for inclusion.

We contacted the authors of each eligible study to obtain deidentified data used for the original analysis, including patient demographics, type of care (inpatient or outpatient), direct and indirect COI, monthly household income, and insurance status. For studies for which individual level data were not available, data were accepted in aggregate form, reporting the average COI by monthly household income quartile or quintile. Although not all studies reported data that would enable calculation of OOP as percent of household income, we also included those that reported total COI and household income because these studies could give an indication of the impact of illness in the absence of insurance and government subsidies. We treated each reported case of influenza as a separate observation (i.e., we did not account for the fact that some patients might have had repeated illnesses, as no personally identifiable information was shared in the data).

### 2.2 Data Analysis

We converted all currencies to US dollars (US$) using the International Monetary Fund official exchange rate for the nominal year [9] and then inflated all results to 2022 US$ using the US Bureau of Economic Analysis gross domestic product (GDP) implicit price deflator [10, 11]. We stratified the reported monthly household incomes in each study into quintiles; if household‐level income data were not shared, we used predetermined quintiles or quartiles as classified by the study authors. Annual income was assumed to be 12 times the reported monthly income. We then calculated the median direct medical cost (payments for medicine and medical services), direct nonmedical cost (other payments required to access healthcare; e.g., travel costs), and indirect cost (loss of income from missed work due to illness), as well as the median total cost (inclusive of direct medical, direct nonmedical, and indirect costs), for each income quintile. We calculated the OOP COI (as reported by the study authors, generally defined as individuals' direct expenses or outlay for health care services [12]) as a proportion of the monthly and annual household income. We used total household income for two reasons: (1) availability of income data across the included studies and (2) to standardize the net proportion of income that is spent on acute respiratory illness. In an ideal scenario, this cost would be calculated using a household's capacity to pay for health care after necessary costs are subtracted from total household income (expenses for housing, transportation, utilities, food, etc.), as indicated by the WHO. However, the availability of household consumption costs was not standard across the studies—the majority only provided data on total household income and the COI.

We reported the median proportion across households (studies from China [13], Colombia [14], and Malaysia [15]), or when only aggregate income strata were provided, we reported the median OOP cost as a proportion of the midpoint household income for each stratum (studies from China [16], Kenya [17], Mongolia [unpublished], South Africa [18], and Thailand [19]). For one study, we reported previously published calculations of the OOP costs as a proportion of household income, as line list data were not available (Bangladesh [20]). We stratified results by household income quintile or quartile and inpatient/outpatient status, population (general population or influenza vaccination target group: children [inclusive of children < 18 years], older adults [≥ 60 or ≥ 65 years, depending on the study], and adults with chronic medical conditions), and World Bank income classification [9]. We only reported results for which there were at least 10 individuals represented in the population. Data from each original study were analyzed separately; no pooled analyses were performed. All analyses were performed in SAS 9.4, and all costs were reported in 2022 US$.

#### 2.2.1 Ethical Clearance

This activity was reviewed by the U.S. CDC, deemed not research, and was conducted consistent with applicable federal law and U.S. CDC policy under project ID 0900f3eb821acfca.

## 3 Results

Of 24 COI studies included in the original systematic review [1], nine reported data on monthly household income and were considered eligible for this analysis. We reviewed an additional 13 studies that were excluded during the systematic review screening phase due to use of an ARI case definition, four of which reported data on household income and were eligible for inclusion. Additionally, an eligible working paper was identified from an unpublished COI study supported by U.S. CDC and conducted in Mongolia (Figure S1).

Of these 14 eligible studies, data from 11 studies (79%) were received and included in this analysis (Tables 1 and S1). Studies represented one low‐income country (Bangladesh [20]), four lower‐middle income countries (El Salvador [21], Kenya [17], Mongolia [unpublished], and Vietnam [22]), and six upper‐middle income countries (China [13, 16], Colombia [14], Malaysia [15], Panama [21], South Africa [18], and Thailand [19]). Of 11 studies, 5 reported COI for the general population, 9 for children, 2 for older adults, and 1 for adults with chronic medical conditions. Five studies reported only costs for hospitalized illnesses, one reported only costs for outpatient illnesses, and five reported both. All studies used a societal perspective.

**TABLE 1:** Characteristics of included cost‐of‐illness studies.

Country	Study	World Bank income group	Case definition	Populations represented				Level of care		Insurance coverage described
				General population	Children	Adults with chronic health conditions	Older adults	Hospitalized	Outpatient	
Bangladesh	Bhuiyan et al. [20]	LIC	LCI	**✓**				**✓**		No public insurance in Bangladesh.
China	Lai et al. [13]	UMIC	ILI		**✓** (< 5 years)	**✓** (18–59 years)	**✓** (≥ 60 years)	**✓**	**✓**	> 95% of the population is covered by insurance
China	Wang et al. [16]	UMIC	LCI		**✓** (< 5 years)			**✓**		54.2% of patients were covered by medical insurance; insurance covered an average of 10% of the cost of care.
Colombia	Salcedo‐Mejia et al. [14]	UMIC	LCI		**✓** (< 18 years)			**✓**		Nearly universal coverage in Colombia; no reported out‐of‐pocket spending on direct medical costs.
El Salvador, Panama	Jara et al. [21]	LMIC (El Salvador); UMIC (Panama)	ARI		**✓** (< 10 years)			**✓**		Health care is free in the public sector (covers 48% of the population in El Salvador; 90% in Panama).
Kenya	Emukule et al. [17]	LMIC	LCI	**✓**	**✓** (< 5 years)			**✓**	**✓**	Hospital costs are covered by the government for children < 5 years; older individuals pay out‐of‐pocket.
Malaysia	Sam et al. [15]	UMIC	ARI		**✓** (< 5 years)			**✓**		56% of households had medical insurance.
Mongolia	Tsogt et al. (unpublished)	LMIC	LCI	**✓**				**✓**	**✓**	87% of outpatients and 88% of inpatients had health insurance.
South Africa	Tempia et al. [18]	UMIC	LCI	**✓**	**✓** (< 5 years)	^a^	^a^	**✓**	**✓**	73% of the societal cost was incurred by the healthcare system and the rest were out‐of‐ pocket expenses.
Thailand	Kittikraisak et al. [19]	UMIC	LCI		**✓** (< 5 years)			**✓**	**✓**	59% of individuals paid out of pocket.
Vietnam	Vo et al., 2017 [22]	LMIC	ILI	**✓**	**✓** (< 14 years)		**✓** (≥65 years)		**✓**	No information reported.

Abbreviations: ARI, acute respiratory infection; ILI, influenza‐like illness; LCI, laboratory‐confirmed influenza; LIC, low‐income country; LMIC, lower‐middle‐income country; UMIC, upper‐middle‐income country.

^a^ Populations represented in dataset, but with insufficient numbers of individuals (fewer than 10) to report results separately.

Among studies that evaluated COI for the general population (inclusive of all ages), only the studies from Kenya and South Africa had data for calculating the median total cost of hospitalized illnesses. By income quintile, this ranged from US$ 100 to 185 in Kenya and US$ 645 to 709 in South Africa. Studies from Kenya, South Africa, and Vietnam had data for calculating the median total cost of outpatient illnesses; these ranged from US$ 8 to 19 in Kenya, US$ 35 to 44 in South Africa, and US$ 53 to 73 in Vietnam (Table 2). OOP expenses, as a proportion of annual household income, were highest among the lowest quintile in all four studies that reported data. For hospitalized illnesses, this proportion was 11% in Kenya, 3% in both Bangladesh and South Africa, and 2% in Mongolia; while OOP expenses for outpatient illnesses accounted for 1% of annual household income in Kenya, South Africa, and Mongolia (Table 2).

**TABLE 2:** Costs of influenza illness and acute respiratory infection among the general population, by hospitalized/outpatient status, country, and household income quintile.

Level of care	Country	Income quintile	Monthly household income bracket (range)	No. cases in quintile	Median direct medical cost^a^	Median direct nonmedical cost^a^	Median indirect cost^a^	Median total cost of illness	Median out‐of‐pocket cost		
			US$		US$	US$	US$	US$	US$	% of monthly HH income	% of annual HH income^b^
Hospitalized	Bangladesh (Bhuiyan)	1	118.29 (median)^c^	8	*NR*	*NR*	*NR*	*NR*	*NR*	37%^d^	3%
		2	148.19 (median)^c^	7	*NR*	*NR*	*NR*	*NR*	*NR*	36%^d^	3%
		3	161.19 (median)^c^	9	*NR*	*NR*	*NR*	*NR*	*NR*	16%^d^	1%
		4	191.08 (median)^c^	9	*NR*	*NR*	*NR*	*NR*	*NR*	34%^d^	3%
		5	337.97 (median)^c^	8	*NR*	*NR*	*NR*	*NR*	*NR*	26%^d^	2%
	Kenya (Emukule)	1	< 68.62^e^	2	149.48	0.00	8.94	158.42	44.50	133%	11%
		2	68.63–137.23^e^	38	65.97	1.81	26.81	99.60	42.22	42%	4%
		3	137.24–343.08^e^	31	73.74	2.68	31.27	103.77	43.56	19%	2%
		4	343.09–686.15^e^	24	83.85	7.57	83.77	185.12	46.18	9%	1%
		5	> 686.16^e^	1	58.31	2.81	67.01	128.14	61.13	6%	1%
	Mongolia (unpublished)	1	167.53 (median)^c^	110	7.94	4.49	*NR*	*NR*	40.12	24%	2%
		2	313.08 (median)^c^	81	6.27	4.18	*NR*	*NR*	40.95	13%	1%
		3	400.82 (median)^c^	95	8.15	4.39	*NR*	*NR*	43.63	11%	1%
		4	559.55 (median)^c^	97	10.45	4.18	*NR*	*NR*	48.06	9%	1%
		5	895.08 (median)^c^	74	12.54	4.18	*NR*	*NR*	54.3	6%	1%
	South Africa (Tempia)	1	< 74.78^e^	8	623.78	6.92	51.32	683.60	17.29	33%	3%
		2	74.87–149.55^e^	23	595.59	5.98	26.17	685.70	17.11	15%	1%
		3	149.65–299.11^e^	14	649.62	6.73	34.02	708.79	14.07	6%	1%
		4	299.20–598.21^e^	14	563.91	6.59	79.59	644.86	14.49	3%	< 1%
		5	> 598.31^e^	9	622.89	7.01	16.73	653.27	18.04	2%	< 1%
Outpatient	Kenya (Emukule)	1	< 68.62^e^	15	5.55	0.00	3.35	8.22	5.55	17%	1%
		2	68.63–137.23^e^	55	5.19	0.00	0.00	11.02	4.71	5%	< 1%
		3	137.24–343.08^e^	64	8.92	0.00	3.91	17.32	5.02	2%	< 1%
		4	343.09–686.15^e^	19	5.04	0.00	0.00	14.61	5.02	1%	< 1%
		5	> 686.16^e^	5	14.35	0.80	6.70	19.44	9.79	1%	< 1%
	Mongolia (unpublished)	1	167.53 (median)^d^	112	11.39	1.25	*NR*	*NR*	12.95	8%	1%
		2	313.08 (median)^d^	95	10.45	0.84	*NR*	*NR*	11.49	4%	< 1%
		3	400.82 (median)^d^	107	8.78	1.42	*NR*	*NR*	10.86	3%	< 1%
		4	559.55 (median)^d^	98	10.45	0.11	*NR*	*NR*	12.16	2%	< 1%
		5	895.08 (median)^d^	55	14.63	0.00	*NR*	*NR*	17.13	2%	< 1%
	South Africa (Tempia)	1	< 74.78^e^	4	23.04	2.48	15.19	40.29	5.42	10%	1%
		2	74.87–149.55^e^	49	25.61	2.43	16.73	44.02	5.33	5%	< 1%
		3	149.65–299.11^e^	21	24.30	2.52	15.70	41.87	5.23	2%	< 1%
		4	299.20–598.21^e^	4	23.93	2.57	14.67	40.34	4.54	1%	< 1%
		5	> 598.31^e^	2	19.16	2.81	12.53	34.49	4.72	< 1%	< 1%
	Vietnam (Vo)	1	< 134.92	132	7.02	29.13	13.49	53.31	*NR*	*NR*	*NR*
		2	134.92–205.07	129	4.86	19.76	21.59	56.43	*NR*	*NR*	*NR*
		3	205.08–215.86	164	5.94	29.10	21.59	62.92	*NR*	*NR*	*NR*
		4	215.87–269.83	102	5.40	16.09	32.38	59.19	*NR*	*NR*	*NR*
		5	> 269.84	131	3.29	16.68	53.97	73.21	*NR*	*NR*	*NR*

*Note:* All costs in 2022 US$.

Abbreviations: HH, household; NR, not reported.

^a^ Specific costs included for each study listed in Table S1.

^b^ Annual income was assumed to be 12 times the reported monthly income.

^c^ Income ranges for each stratum were not stated; medians are shown.

^d^ Aggregate estimates of out‐of‐pocket costs as a proportion of monthly household income obtained from publication.

^e^ Income strata precalculated by study authors; midpoints used for calculation of median OOP costs as a proportion of monthly household income.

Among studies that estimated the COI for children, the median total cost of hospitalized illnesses, by income strata, ranged from US$ 482 to 1025 in China, US$ 873 to 2669 in Colombia, US$ 46 to 57 in El Salvador, US$ 59 to 100 in Panama, US$ 103 to 170 in Kenya, US$ 319 to 377 in Malaysia, US$ 644 to 696 in South Africa, and US$ 131 to 1091 in Thailand (Table 3). The median total cost of outpatient illnesses by strata was US$ 77–128 in China, US$ 6–151 in Kenya, US$ 28–49 in South Africa, US$ 19–31 in Thailand, and US$ 78–308 in Vietnam. OOP expenses, as a proportion of annual household income, were highest among the lowest quintile in six of seven studies that reported data (all except Colombia). Among the lowest income stratum, OOP expenses for hospitalized illnesses represented 11% of annual household income in Kenya, 9% and 5% in China, 5% in Thailand, 3% in South Africa, 2% in Malaysia, and 1% in Colombia; OOP expenses for outpatient illnesses represented 3% of annual income in Kenya and 1% across China, South Africa, and Thailand (Table 3).

**TABLE 3:** Costs of influenza illness and acute respiratory infection among children,^a^ by hospitalized/outpatient status, country, and household income quintile.

Level of care	Country	Income quintile	Monthly household income bracket (range)	No. cases in quintile	Median direct medical cost^b^	Median direct nonmedical cost^b^	Median indirect cost^b^	Median total cost of illness	Median out‐of‐pocket cost		
			US$		US$	US$	US$	US$	US$	% of monthly HH income	% of annual HH income^c^
Hospitalized	China (Lai)	1	< 634.69	77	458.43	15.81	0.00	481.70	277.50	58%	5%
		2	634.85–952.03	80	641.89	34.53	17.63	685.43	362.71	46%	4%
		3	952.19–1586.72	135	764.06	55.32	49.36	886.97	640.87	40%	3%
		4	1586.88–2062.73	18	869.01	107.00	79.24	1137.69	740.81	37%	3%
		5	> 2062.89	70	802.82	55.43	58.18	1024.96	600.44	19%	2%
	China (Wang)	1	< 1401.02^d^	128	880.79	60.53	*NR*	*NR*	773.69	110%	9%
		2	1401.02–1961.40^d^	143	1021.79	67.25	*NR*	*NR*	896.00	53%	4%
		3	2101.62–2661.99^d^	17	1055.12	84.06	*NR*	*NR*	1136.12	48%	4%
		4	2802.04–3362.59^d^	12	758.42	101.72	*NR*	*NR*	750.98	24%	2%
		5	> 3502.64^d^	15	784.95	100.88	*NR*	*NR*	762.01	22%	2%
	Colombia (Salcedo‐Mejia)	1	< 381.65	3	2043.39	*NR*	123.8	2202.76	35.57	10%	1%
		2	381.65–422.03	2	2347.18	*NR*	191.89	2575.58	36.51	9%	1%
		3	422.04–512.48	4	2335.52	*NR*	216.65	2668.65	70.78	16%	1%
		4	512.49–738.57	3	711.89	*NR*	99.04	873.27	46.42	7%	1%
		5	> 738.58	3	1619.10	*NR*	148.56	1906.65	126.61	16%	1%
	El Salvador (Jara)	1	< 214.85^d^	74	24.86	22.10	29.47	46.42	*NR*	*NR*	*NR*
		2	216.09–429.71^d^	74	23.94	22.41	29.83	45.92	*NR*	*NR*	*NR*
		3	430.93–613.87^d^	42	46.65	23.64	24.55	57.21	*NR*	*NR*	*NR*
		4	> 615.09^d^	79	26.29	24.55	36.83	46.65	*NR*	*NR*	*NR*
	Panama (Jara)	1	< 214.85^d^	28	13.51	27.93	35.91	58.62	*NR*	*NR*	*NR*
		2	216.09–429.71^d^	54	27.81	25.48	24.55	64.85	*NR*	*NR*	*NR*
		3	430.93–613.87^d^	73	52.15	56.72	103.75	100.06	*NR*	*NR*	*NR*
		4	> 615.09^d^	88	46.65	52.79	71.21	123.7	*NR*	*NR*	*NR*
	Kenya (Emukule)	1	< 68.62^d^	2	149.48	0.00	8.94	158.42	44.50	133%	11%
		2	68.63–137.23^d^	32	65.97	2.15	26.81	103.49	36.19	36%	3%
		3	137.24–343.08^d^	26	72.00	2.21	38.53	102.56	41.55	18%	2%
		4	343.09–686.15^d^	21	82.85	6.03	83.77	170.44	45.57	9%	1%
		5	> 686.16^d^	0	NA	NA	NA	NA	NA	NA	NA
	Malaysia (Sam)	1	< 1096.59	33	194.46	36.55	114.7	376.78	235.4	28%	2%
		2	1096.60–1279.35	51	168.14	40.21	114.7	335.63	219.5	18%	2%
		3	1279.36–1571.77	32	171.25	42.04	114.7	318.87	222.43	15%	1%
		4	1571.78–1827.64	41	171.8	49.35	114.7	336.65	218.95	13%	1%
		5	> 1827.65	37	177.65	36.55	159.22	355.29	214.93	8%	1%
	South Africa (Tempia)	1	< 74.78^d^	4	649.95	5.71	19.35	674.16	15.47	30%	3%
		2	74.87–149.55^d^	10	662.01	6.17	17.76	696.17	17.30	15%	1%
		3	149.65–299.11^d^	6	641.96	8.88	18.88	668.92	18.27	8%	1%
		4	299.20–598.21^d^	4	632.70	1.68	18.88	654.30	13.88	3%	< 1%
		5	> 598.31^d^	7	622.89	7.01	16.73	643.83	18.04	2%	< 1%
	Thailand (Kittikraisak)	1	< 348.70^d^	1	87.09	14.80	29.60	131.49	101.89	58%	5%
		2	348.74–697.44^d^	8	285.22	22.59	35.51	325.23	190.60	36%	3%
		3	697.48–1046.18^d^	1	239.09	9.53	0.00	248.61	NA^e^	NA^e^	NA^e^
		4	1046.21–1394.92^d^	4	154.87	17.56	0.00	171.07	221.67	18%	2%
		5	> 1394.95^d^	1	667.11	53.88	370.34	1091.33	720.99	52%	4%
Outpatient	China (Lai)	1	< 793.36	514	53.63	3.17	0.00	76.87	62.67	10%	1%
		2	793.52–1269.37	485	62.63	3.95	0.00	85.22	69.70	6%	1%
		3	1269.53–1586.72	521	64.65	4.15	0.00	100.21	79.17	5%	< 1%
		4	1586.88–2856.09	290	55.90	3.19	0.00	87.09	61.09	3%	< 1%
		5	> 2856.25	449	76.41	7.93	0.00	127.61	79.81	3%	< 1%
	Kenya	1	< 68.62^d^	4	7.74	1.34	4.47	15.45	10.43	31%	3%
		2	68.63–137.23^d^	31	5.23	0.00	0.00	8.85	4.32	4%	< 1%
		3	137.24–343.08^d^	55	9.12	0.00	7.82	17.96	5.47	2%	< 1%
		4	343.09–686.15^d^	13	5.73	0.00	0.00	5.73	5.04	1%	< 1%
		5	> 686.16^d^	2	16.49	0.94	134.03	151.46	10.86	1%	< 1%
	South Africa (Tempia)	1	< 74.78^d^	1	28.13	2.24	18.88	49.26	5.42	10%	1%
		2	74.87–149.55^d^	17	26.73	2.52	15.70	47.11	5.42	5%	< 1%
		3	149.65–299.11^d^	7	25.05	2.43	15.70	44.02	5.23	2%	< 1%
		4	299.20–598.21^d^	2	27.81	2.57	14.67	45.06	5.52	1%	< 1%
		5	> 598.31^d^	1	13.83	1.87	12.53	28.23	2.80	< 1%	< 1%
	Thailand (Kittikraisak)	1	< 348.70^d^	6	8.67	4.55	0	19.18	12.57	7%	1%
		2	348.74–697.44^d^	49	8.62	9.34	0	28.31	18.5	4%	< 1%
		3	697.48–1046.18^d^	21	10.13	7.4	0	21.16	13.34	2%	< 1%
		4	1046.21–1394.92^d^	16	6.21	7.18	15.99	29.04	11.62	1%	< 1%
		5	> 1394.95^d^	35	7.24	11.43	12.57	30.86	18.76	1%	< 1%
	Vietnam (Vo)	1	< 134.92	46	6.62	59.06	10.79	77.75	*NR*	*NR*	*NR*
		2	134.92–205.07	22	5.75	74.42	24.29	107.91	*NR*	*NR*	*NR*
		3	205.08–215.86	21	13.49	74.91	21.59	103.89	*NR*	*NR*	*NR*
		4	215.87–269.83	2	5.08	31.82	43.00	79.89	*NR*	*NR*	*NR*
		5	> 269.84	7	37.72	192.01	71.96	308.17	*NR*	*NR*	*NR*

*Note:* All costs in 2022 US$.

Abbreviations: HH, household; NA, not applicable (no observations in strata); NR, not reported.

^a^ Inclusive of children with chronic medical conditions (not reported separately).

^b^ Specific costs included for each study listed in Table S1.

^c^ Annual income was assumed to be 12 times the reported monthly income.

^d^ Income strata precalculated by study authors; midpoints used for calculation of median out‐of‐pocket costs as a proportion of monthly household income.

^e^ OOP costs not reported by the sole individual in this stratum.

Lastly, among studies examining the COI for older adults, the median total cost of hospitalized illnesses, by income quintile, ranged from US$ 164 to 493 in China; the median total cost of outpatient illnesses was US$ 15–32 in China and US$ 20–111 in Vietnam (Table 4). In the one study that examined the COI for adults with chronic medical conditions (China), the median total cost of hospitalized illness, by income quintile, ranged from US$ 74 to 505, and outpatient illnesses ranged from US$ 21 to 44. Again, for both older adults and adults with chronic medical conditions, OOP costs as a proportion of annual household income were highest among the lowest income quintile in all studies; the proportion was 85% for hospitalized illnesses and 2% for outpatient illnesses for all older adults in China and 59% for hospitalizations and 1% for outpatient illnesses for adults with chronic medical conditions in China (Table 4).

**TABLE 4:** Costs of influenza illness and acute respiratory infection among older adults and adults with chronic medical conditions, by hospitalized/outpatient status, country, and household income quintile.

Level of care	Country	Income quintile	Monthly household income bracket (range)	No. cases in quintile	Median direct medical cost^a^	Median direct nonmedical cost^a^	Median indirect cost^a^	Median total cost of illness	Median out‐of‐pocket cost		
			US$		US$	US$	US$	US$	US$	% of monthly HH income	% of annual HH income^b^
Older adults^c^											
Hospitalized	China (Lai)	1	< 41.25	20	631.49	0.00	0.00	633.09	163.77	1022%	85%
		2	41.41–253.87	20	815.98	11.90	14.77	917.86	163.53	108%	9%
		3	254.03–476.02	19	1279.03	7.94	10.58	1332.88	492.83	155%	13%
		4	476.17–952.03	20	431.30	3.96	50.51	581.79	219.16	35%	3%
		5	> 1110.86	19	704.88	7.94	52.89	789.54	314.41	17%	1%
Outpatient	China (Lai)	1	< 158.67	54	16.08	0.00	0.00	19.14	15.44	23%	2%
		2	158.83–396.68	42	30.92	0.00	0.00	34.45	27.40	9%	1%
		3	396.84–793.36	60	39.67	0.00	0.00	53.76	32.23	5%	< 1%
		4	793.52–1269.37	37	40.40	0.00	0.00	48.20	27.82	3%	< 1%
		5	> 1269.53	44	33.78	0.00	0.00	44.69	21.05	1%	< 1%
	Vietnam (Vo)	1	< 134.92	5	2.91	10.46	13.49	19.59	*NR*	*NR*	*NR*
		2	134.92–205.07	1	2.43	7.56	10.79	20.78	*NR*	*NR*	*NR*
		3	205.08–215.86	5	7.56	11.44	21.59	45.33	*NR*	*NR*	*NR*
		4	215.87–269.83	3	7.56	22.13	48.57	110.63	*NR*	*NR*	*NR*
		5	> 269.84	3	10.96	25.10	21.59	57.64	*NR*	*NR*	*NR*
Adults with chronic medical conditions											
Hospitalized	China (Lai)	1	< 95.20	5	784.58	3.17	9.52	802.83	505.01	707%	59%
		2	95.36–317.34	7	607.29	31.67	21.16	766.18	183.88	64%	5%
		3	317.50–634.69	4	706.42	18.66	82.86	871.89	278.51	54%	5%
		4	634.85–1110.70	4	275.67	1.11	91.24	354.12	73.50	9%	1%
		5	> 1110.86	5	1309.09	40.82	0.00	1309.09	489.70	31%	3%
Outpatient	China (Lai)	1	< 317.34	37	33.62	0.00	0.00	36.60	26.13	12%	1%
		2	317.50–634.69	36	23.36	0.00	0.00	30.19	21.17	4%	< 1%
		3	634.85–1110.70	38	24.93	0.00	0.00	59.48	28.65	3%	< 1%
		4	1110.86–1586.72	39	40.40	0.00	0.00	48.48	21.22	1%	< 1%
		5	> 1586.88	28	71.01	0.95	0.00	97.77	44.29	2%	< 1%

*Note:* All costs in 2022 US$.

Abbreviations: HH, household; NR, not reported.

^a^ Specific costs included for each study listed in Table S1.

^b^ Annual income was assumed to be 12 times the reported monthly income.

^c^ Included adults aged ≥ 60 years (China) and ≥ 65 years (Vietnam).

## 4 Conclusion

Our findings document that influenza illness and ARIs disproportionately affect the lowest income households. Our results suggest that expenses from these illnesses, particularly ones that result in hospitalizations, could be catastrophic for low‐income households. We documented several instances where median OOP costs exceeded 10% of annual household income, a commonly accepted definition for CHE: hospitalized illnesses among the general population and children in Kenya [17], as well as older adults and adults with chronic medical conditions in China [13].

In cases where expenses from influenza illness and ARIs did not meet the definition of catastrophic based on the calculated proportion of annual household income, OOP expenses may have the potential to impact the financial well‐being of the household over a shorter time period. For example, in households with little savings, OOP costs may impede the ability to pay for food and other basic services in the short term. The Bangladesh COI study, in which median OOP expenses were 37% of monthly income of households in the lowest income quintile, reported that 100% of these households had to borrow money to cover OOP expenses from hospitalizations [20]. Other mechanisms for addressing OOP costs, in addition to borrowing, include diversifying income, selling assets, or reducing household expenditures, most often on food [23, 24], which can have additional negative downstream effects.

Among the studies that evaluated multiple target groups, OOP expenses among hospitalized cases as a proportion of income were particularly high among the lowest income stratum for older adults and adults with chronic medical conditions in China [13]; they were also high (11%) in children and in the general population in Kenya [17]. In the China study, drivers of these high costs included nonreimbursed hospitalization expenses, direct nonmedical costs, and over‐the‐counter medicines, as well as low incomes of the populations in these groups [13]. In the Kenya study, children comprised the majority of the population, they were also hospitalized longer, and their caregivers were more likely to buy over‐the‐counter medicine before being admitted, which likely drove the higher costs.

Our findings are consistent with a 2019 systematic review of 44 studies from 22 countries [25] and a 2022 systematic review of studies in Sub‐Saharan African countries [26], which both found that low economic status, hospitalization, older age, and chronic illness were associated with household CHEs from all causes. Both reviews, as well as a 2003 review of CHEs in 59 countries [5], indicated that lack of health insurance was associated with CHEs; although we could not assess the impact of insurance on illness‐associated expenditures in this analysis because coverage and schemes varied widely, this topic merits further exploration in future studies.

Evaluating household financial hardship solely based on OOP expenditures does not take into account the effect of indirect costs, such as loss of wages due to missing work, which can be substantial in low‐income households and in LMICs [1, 2]. The studies included in this review indicate that the total COI can inflict a financial burden on households that is several folds higher than the OOP costs alone. Additionally, individuals with respiratory infections might incur long‐lasting economic effects beyond the course of acute illness (e.g., from missed education or disability), which could further impoverish households and communities. Alternatively, individuals may not seek care if they know they would not be able to afford treatment.

These findings are subject to several limitations. Although the included studies were identified through a systematic search, they might not be representative of all available literature on influenza and ARI COI; we only included studies for which authors agreed to share data and additionally included one unpublished study based on prior engagement with CDC. There was wide variation in methodology (e.g., in the cost components included in COI) and illness case definitions used across studies, limiting the ability to synthesize results across studies. Relatedly, characteristics of each study setting, such as public and private insurance schemes and coverage, likely varied substantially. Economically disadvantaged households might be less likely to seek care because of financial and social barriers [27], and thus, case recruitment and cost estimates might be selective to only those who can afford to seek care. The generalizability of results might be limited by the small number of households within some income quintiles, and for some studies, only aggregate data were available.

All but one of the included studies represented middle income countries; further evaluation of cost disparities within low‐ and high‐income countries is warranted. We restricted our search to studies that used a case definition of laboratory‐confirmed influenza or syndromic definitions requiring respiratory symptoms (ILI, SARI, and ARI); however, influenza can also present as nonrespiratory illness with broader consequences than those here analyzed, and thus, the true economic impact might be underestimated [28]. Although we retrieved studies using an ARI case definition from the screening phase of the original systematic review search [1], the search terms in the systematic review were not designed to capture ARI studies, and some literature might have been missed. As previously discussed, cost data were reported for illness among a single household member; however, a household might incur multiple influenza illnesses during a year and could incur higher costs than captured in this analysis.

We conducted this analysis under several assumptions. Due to data availability, we estimated the potential for CHEs based on total reported household income, which might overestimate actual income available to spend on healthcare after subsistence expenditures. We also assumed stable incomes for households when calculating the annual income as 12 times the reported monthly income; however, this might not be true for all households, particularly those in settings with large informal labor markets. We used the indicator of 10% of total household income to define CHE, rather than the WHO indicator of 40% of a household's capacity to pay for health care. This proxy may underestimate the impacts on lower income households, which typically spend a greater proportion of their income on housing, food, and utilities than higher income households—therefore, spending 10% of total household income on OOP health care costs may equate to greater than 40% of a household's capacity to pay for health care in low‐income households. Considering the “capacity to pay for health care” indicator, spending 10% of total household income on OOP costs may not be catastrophic for some households but will exceed catastrophic levels in others. Although we stratified our analysis by household income quintile, this does not adequately account for a household's ability to pay for health care and thus may introduce bias into the costs that are considered catastrophic, particularly for lower income quintiles.

In summary, we documented the potential for households to incur CHEs from influenza illness and ARIs, particularly among the lowest income strata and in cases of hospitalized illness. Interventions to prevent and reduce severity of influenza illness, such as seasonal influenza vaccination, as well as other interventions to reduce ILI and ARI, such as nonpharmaceutical interventions, antivirals, and other respiratory vaccines (e.g., respiratory syncytial virus and COVID‐19 vaccines), could have significant benefits in reducing COI. The disparate impact of influenza illness and ARIs on households and risk for CHEs also underscores the global importance of health financing policies that reduce financial hardship from illness [7].

Future economic evaluations for influenza and ARI COI should consider including questions on household income and on mechanisms employed to cope with high health expenditures to contextualize the impact and report findings. From the 11 studies we included in this analysis, only one reported costs stratified by household income in the results [20]; additional efforts to examine COI by income level, taking into account the country‐specific context, would strengthen the global evidence base. Additionally, future evaluations of the cost‐effectiveness of interventions (e.g., vaccination) could incorporate health inequities, such as those stemming from income disparities, into their analyses by using equity‐informative methods such as extended or distributional cost‐effectiveness [29, 30, 31]. Ultimately, continuing to evaluate and mitigate the costs of influenza illness, particularly among households in the lowest income strata, is a matter of health equity.

## Author Contributions

**Natalie Wodniak:** investigation, formal analysis, writing – original draft, methodology, writing – review and editing, data curation. **Radhika Gharpure:** investigation, formal analysis, writing – original draft, methodology, writing – review and editing, conceptualization, data curation. **Luzhao Feng:** investigation, formal analysis, writing – review and editing, data curation. **Xiaozhan Lai:** investigation, formal analysis, writing – review and editing, data curation. **Hai Fang:** investigation, formal analysis, writing – review and editing, data curation. **Jianmei Tian:** investigation, formal analysis, writing – review and editing, data curation. **Tao Zhang:** investigation, formal analysis, writing – review and editing, data curation. **Genming Zhao:** investigation, formal analysis, writing – review and editing, data curation. **Fernando Salcedo‐Mejía:** investigation, formal analysis, writing – review and editing, data curation. **Nelson J. Alvis‐Zakzuk:** investigation, formal analysis, writing – review and editing, data curation. **Jorge Jara:** investigation, formal analysis, writing – review and editing, data curation. **Fatimah Dawood:** investigation, formal analysis, writing – review and editing, data curation. **Gideon O. Emukule:** investigation, formal analysis, writing – review and editing, data curation. **Linus K. Ndegwa:** formal analysis, investigation, writing – review and editing, data curation. **Ching Sam:** investigation, formal analysis, writing – review and editing, data curation. **Tsogt Mend:** investigation, formal analysis, writing – review and editing, data curation. **Baigalmaa Jantsansengee:** investigation, formal analysis, writing – review and editing, data curation. **Stefano Tempia:** investigation, formal analysis, writing – review and editing, data curation. **Cheryl Cohen:** investigation, formal analysis, writing – review and editing, data curation. **Sibongile Walaza:** investigation, formal analysis, writing – review and editing, data curation. **Wanitchaya Kittikraisak:** investigation, formal analysis, writing – review and editing, data curation. **Arthorn Riewpaiboon:** investigation, formal analysis, writing – review and editing, data curation. **Kathryn E. Lafond:** investigation, formal analysis, writing – review and editing, conceptualization, data curation. **Nelly Mejia:** investigation, formal analysis, writing – review and editing, conceptualization, methodology, writing – original draft, data curation. **William W. Davis:** conceptualization, investigation, formal analysis, writing – review and editing, supervision, methodology, project administration, resources, data curation.

## Conflicts of Interest

CC has received grant funds from the U.S. CDC to the institution in support of this work. She has also received granted funds to the institution unrelated to this work from Sanofi Pasteur, Wellcome Trust, Bill and Melinda Gates Foundation, and the Taskforce for Global Health. The other authors have no conflicts to report. The findings and conclusions in this report are those of the authors and do not necessarily represent the official position of the U.S. Centers for Disease Control and Prevention.

## Supporting information


**Table S1** Additional methodological characteristics of included cost‐of‐illness studies.

**Figure S1** PRISMA flow diagram of study selection process.

## Data Availability

The data that support the findings of this study are available from the authors upon reasonable request.
