# Supplementary material for: Costs of Influenza Illness and Acute Respiratory Infections by Household Income Level: Catastrophic Health Expenditures and Implications for Health Equity
Source: Influenza Other Respir Viruses. 2025 Jan 9;19(1):e70059. doi: 10.1111/irv.70059 (PMC11718101; doi:10.1111/irv.70059)
Supplement: Supplementary file 1 — Table S1 Additional methodological characteristics of included cost‐of‐illness studies. [file IRV-19-e70059-s001.docx]

**Supplementary Table 1: Additional methodological characteristics of included cost-of-illness studies.**

| **Study** | **Country** | **Data source; public or private facilities** | **Level (national/sub-national) represented by sample** | **Study period** | **Direct medical costs included** | **Direct non-medical costs included** | **Indirect costs included** | **Original currency reported** |
| --- | --- | --- | --- | --- | --- | --- | --- | --- |
| Bhuiyan et al., 2014 ^20^ | Bangladesh | 4 hospitals; public and private | National | 2010 | Physician consultation, hospital bed, medicines, diagnostic tests | Food, lodging, travel | Lost income from patients and caregivers | 2010 US$ |
| Lai et al., 2021 ^13^ | China | 148 community health centers; public | National | 2019 | Outpatient service, inpatient  service, over-the-counter medications | Transportation, nutrition/food, accommodation,  nursing-worker hiring | Lost income  from patients and families | 2020 CNY |
| Wang et al., 2021 ^16^ | China | 1 hospital; not reported | Sub-national (facility) | 2011–17 | Pharmaceuticals, laboratory tests, therapeutics, materials, radiology, registration, bed costs, nursing | Transportation, accommodation, additional  nourishment | Lost income from caregivers | 2017 US$ |
| Salcedo-Mejia et al., 2019 ^14^ | Colombia | 1 hospital; not reported | Sub-national (facility) | 2014 | Consultation, bed cost, laboratory and images, materials and medical supplies, drugs, procedures | Transportation, food | Lost income from caregivers | 2014 US$ |
| Jara et al., 2019 ^21^ | El Salvador, Panama | 3 hospitals (El Salvador); 2 hospitals (Panama); all public | National | 2012–13 | Outpatient consultation, medications, hospital fees | Transportation, childcare | Lost income from caregivers | 2013 US$ |
| Emukule et al., 2019 ^17^ | Kenya | 4 hospitals and 1 clinic; public and private | National | 2013–14 | Medications, laboratory investigations, hospital bed fees, hospital  management costs | Transportation | Lost income  from patients and families | 2014 US$ |
| Sam et al., 2021 ^15^ | Malaysia | 1 public hospital | Sub-national (facility) | 2013–15 | Hospital bills, primary care and non-medical practitioner consultations, medications, laboratory tests | Transportation | Lost income from caregivers | 2014 US$ |
| Tsogt et al. (unpublished) | Mongolia | 7 hospitals and 10 clinics; not reported | National | 2018–19 | Medications, diagnostics, consultation costs | Transportation, food, phone charges | Lost income from patients and caregivers | 2019 MNT |
| Tempia et al., 2019 ^18^ | South Africa | 7 hospitals and 2 clinics; public | National | 2013–15 | Facility fee, consultation,  ICU, chest X‐ray, oxygen therapy, medications,  laboratory tests | Transportation, additional food | Lost income from patients and caregivers | 2015 US$ |
| Kittikraisak et al., 2018 ^19^ | Thailand | 1 hospital; public | Sub-national (facility) | 2011–15 | Medicines, laboratory tests, other healthcare costs | Transportation | Lost income from employed primary and secondary caregivers, opportunity cost from unemployed primary and secondary caregivers | 2015 US$ |
| Vo et al., 2017 ^22^ | Vietnam | 15 pharmacies (private), 3 clinics (private), and 1 hospital (public) | Sub-national (region) | 2016 | Diagnosis, therapeutics, prescriptions, drugs, hospital services | Transportation, meals, accommodation, caregiver costs | Lost income from patients | 2016 US$ |

Abbreviations: CNY, Chinese yuan renminbi; MNT, Mongolian tugrik; US$, U.S. dollars.
