# Supplementary material for: Costs of Influenza Illness and Acute Respiratory Infections by Household Income Level: Catastrophic Health Expenditures and Implications for Health Equity
Source: Influenza Other Respir Viruses. 2025 Jan 9;19(1):e70059. doi: 10.1111/irv.70059 (PMC11718101; doi:10.1111/irv.70059)
Supplement: Supplementary file 2 — Figure S1 PRISMA flow diagram of study selection process. [file IRV-19-e70059-s002.docx]

Records identified from:

- Embase (n=3,845)
- Medline (n=3,675)
- Scopus (n=1,950)
- CINAHL (n=334)
- Cochrane (n=326)

Total (**n=10,130**)

Duplicate records removed before screening

(n=3,516)

**Identification**

Records manually excluded for failure to meet inclusion criteria

(n=6,431)

Titles/abstracts screened

(**n=6,614**)

**Screening**

**Original systematic review (published in Gharpure et al., 2024)**

Full texts excluded (n=134):

- Not low- or middle-income setting (n=33)
- Evaluating cost-effectiveness of non-vaccine interventions or comparing vaccine products (n=30)
- No economic findings (n=29)
- Not original, peer-reviewed results (n=17)
- Not laboratory-confirmed influenza or ILI/SARI (n=13)
- Pandemic/novel influenza or 2009 results could not be disaggregated (n=10)
- Not population-level program costing (n=2)

Full texts assessed for eligibility

(**n=183**)

Met inclusion criteria (**n=49**)

*1 study added from review of references from included studies*

**Included**

Included studies (**n=50**)

**Included**

Data received and study included in analysis (**n=11**)

**Screening/outreach**

Cost-of-illness studies reporting data on household income (**n=9**)

Cost-of-illness studies reporting data on household income (**n=4**)

+

*1 unpublished study supported by US CDC added*

Lead authors contacted for deidentified data from study (**n=14**)

+

**Identification**

Cost-of-illness studies originally excluded for non-ILI/SARI case definition (**n=13**)

Cost-of-illness studies included in systematic review (**n=24**)

**Secondary analysis of cost-of-illness literature**
